# Development of a Reliable Analytical Method for Liquid Anion-Exchange Extraction and Separation of Neodymium(III)

**DOI:** 10.1100/2012/856948

**Published:** 2012-06-04

**Authors:** Balasaheb N. Kokare, Ganesh S. Kamble, Balasaheb M. Sargar, Mansing A. Anuse

**Affiliations:** ^1^Department of Chemistry, Raje Ramrao Mahavidyalaya, Jath, Sangli 416 404, India; ^2^Analytical Chemistry Laboratory, Department of Chemistry, Shivaji University, Kolhapur 416 004, India; ^3^Analytical Chemistry Laboratory, PG Department of Chemistry, Jaysingpur College, Jaysingpur, Kolhapur 416 116, India

## Abstract

The liquid-liquid extraction of neodymium(III) from succinate media (0.06 M) has been studied at pH 6.0 with the solution of 0.1 M of N-*n*-octylaniline in xylene when equilibrium is maintained for 5 min. The back-extraction of neodymium(III) has been performed by using 0.1 M HClO_4_. The effect of various parameters, such as pH, equilibrium time, extractant concentration, stripping agents, organic diluents, and aqueous to organic volume ratio on the extraction of neodymium(III) has been studied. On the basis of slope analysis, the stoichiometry of the extracted species was determined as 1 : 1 : 2 [RR′NH_2_^+^Nd(succinate)_2_^−^]_(org)_. The method is free from interference of large number cations and anions. The method was used for the selective extraction of neodymium(III) from its binary mixture with U(VI), Zr(IV), Nb(V), La(III), Th(IV), Ce(IV), and Y(III). The proposed method is selective and was successfully applied to the synthetic mixtures to show the practical utility of the extractant.

## 1. Introduction

Neodymium is never found in nature as the free element; rather, it occurs in ores such as monazite sand and bastnasite (40 ppm by weight) [1] that contain small amounts of all the rare earth metals. Neodymium can also be found in misch metal; it is difficult to separate from other rare earth elements.

 Neodymium magnets are the strongest permanent magnets known: Nd_2_Fe_14_B [2]. Neodymium magnets appear in the products such as microphones, professional loudspeakers, inear headphones, guitar and bass guitar pick-ups, and computer hard disc where low-mass small volume or strong magnetic fields are required. It exists in two allotropic forms [3]. Neodymium colours glass in delicate shades ranging from pure violet through wine-red and warm gray. Neodymium salts are used as a colourant for enamels. Probably because of similarities to Ca(II), Nd(III) has been reported to promote plant growth [4]. Rare-earth-element compounds are frequently used in China as fertilizers. Samarium-Neodymium dating is useful for determining the age relationship of rocks and meteorites. Certain transparent materials with a small concentration of neodymium ion can be used in laser as gain media for infrared wavelengths. Neodymium compounds are of low- to moderate-toxicity. Neodymium dusts and salts are very irritating to the eye, mucous membranes, and moderately irritating to skin. Breathing the dust can cause lung embolisms and accumulated exposure damages the liver. It also acts as an anticoagulant especially when given intravenously.

 Solvent extraction is one of the efficient methods for separation technology because of its simplicity, speed and applicability to both tracer and macro amounts of metal ions. As there are a number of different solvent extraction systems that could be used for metal ions separation, synergistic extraction systems have received attention for a long time. The synergistic solvent extraction of multicharged transition ions as well as lanthanide and actinide ions has been extensively studied using various chelating or complexing agents such as **β**-diketones, high-molecular-weight amines, amine salts, crown ethers, quaternary ammonium salts- and phosphorus-containing compounds, as synergists. It has been found that the metal ions can be extracted synergistically with considerable enhancement [5]. A systematic study of the synergistic solvent extraction and separation of neodymium(III) with 1-phenyl-3-methyl-4-benzoyl pyrazol-5-one in the presence of the quaternary ammonium salt, aliquat 336 [5, 6], 4-benzoyl-3-phenyl-5-isoxazolone alone and quaternary ammonium salt, aliquat 336 [7], *bis*(2,4,4,-trimethylpentyl) dithiophosphinic acid and trialkylphosphine oxide [8] were studied. However, methods require more than 1 h equilibration time for the quantitative extraction. Extraction behavior of neodymium(III) with some selected substituted malonamides [9, 10], *n*-octyl(phenyl)-*N*-*N*-diisobutylcarbamoyl methylphosphine oxide and mixed solvent using amino polyacetic acid and diethylene triamine pentaacetic acid nitrate solution [11], a mixture of calyx[4]arene carboxyl derivative and primary amine N1923 [12] has been investigated. Extraction depends on the concentration of salting out agents. A synergistic solvent extraction of neodymium(III) with thenoyltrifluoroacetone and aliquat 336 [13, 14], mixture of di-(2-ethylhexyl) phosphoric acid and S-octylphenoxyacetic acid [15], PC88A, and saponified PC88A [16] have been studied. The extraction was carried from mineral acid media; hence the methods are not eco-friendly. A separation process of neodymium(III) by liquid-liquid extraction with *bis*(2-ethylhexyl) phosphinic acid [17] was investigated. The 10 stages of extraction followed by 5 stages of scrubbing were considered.

 There are very few methods reported for neodymium(III) by using high-molecular-weight amine. Extraction of trivalent neodymium by high-molecular-weight amine such as decylamine [18] from 0.5 to 3 M nitric acid solution containing potassium phosphotungstate has been investigated. Desai and Shinde reported liquid anion exchange extraction and separation of neodymium(III) from sodium succinate solution with tri-*n*-octylamine [19]. However, the method requires high reagent concentration in order to ensure the complete extraction. In this communication, systematic studies on the extractive properties of N-*n*-octylaniline in xylene from succinate media have been proposed.

## 2. Experimental

### 2.1. Apparatus

An Elico digital spectrophotometer model SL-171 with 1 cm quartz cells was used for absorbance measurements. pH measurements were carried out using an Elico digital pH meter model LI-120.

Glass vessels were cleaned by soaking in acidified solution of potassium dichromate followed by washing with soap and water, and rinsed two times with water.

All the apparatus were used during the time period from May 2009 to July 2009.

### 2.2. Reagents

#### 2.2.1. Standard Neodymium(III) Solution

The stock solution of neodymium(III) was prepared by dissolving 0.294 g of neodymium oxide in 10 mL of perchloric acid and diluted with 250 mL of water. The solution was standardized [20] and working solution was prepared as required (60 *μ*g/mL) by appropriate dilution of the stock solution.

#### 2.2.2. 4-(2-Pyridylazo)-Resorcinol (PAR) Solution (0.05% w/v)

A 0.05% aqueous solution of 4-(2-pyridylazo)-resorcinol was prepared in water and used for the spectrophotometric determination of neodymium(III). 

#### 2.2.3. N-n-Octylaniline

The extractant N-*n*-octylaniline was prepared by the method of Gardlund et al. [21] and its solutions were prepared in xylene. All other chemicals used in this work were of AR grade. Double distilled water was used throughout the work.

#### 2.2.4. General Extraction and Determination Procedure for Neodymium(III)

An aliquot of 60 *μ*g neodymium(III) solution was mixed with a sufficient quantity of sodium succinate (0.41 g) to make its concentration 0.06 M in a total volume of 25 mL of the solution. The pH of the aqueous solution was adjusted from 6.0 to 7.5 by dilute sodium hydroxide and perchloric acid solution. The solution was then transferred to a 125 mL separating funnel and shaken with 10 mL of 0.1 M N-*n-*octylaniline in xylene for 5 min. After separating the two phases, the aqueous phase was discarded and the organic phase was stripped with 2 × 5 mL portions of 0.1 M perchloric acid solution. The stripped aqueous phase was equilibrated with 5 mL of xylene to remove the traces of dissolved amine and evaporated to moist dryness. The slightly acidic solution was transferred to a 25 mL volumetric flask followed by 1 mL of 0.05% 4-(2-pyridylazo)-resorcinol solution, the pH was adjusted to 6.00 with dilute sodium hydroxide and perchloric acid solution. The solution was diluted to 25 mL with distilled water and the absorbance after 30 min of orange-red complex at 515 nm read was spectrophotometrically [22] against the reagent blank in the reference cell. The amount of neodymium(III) in the solution was calculated from the calibration curve.

## 3. Results and Discussion

### 3.1. Extraction as a Function of pH

**Figure 1:**
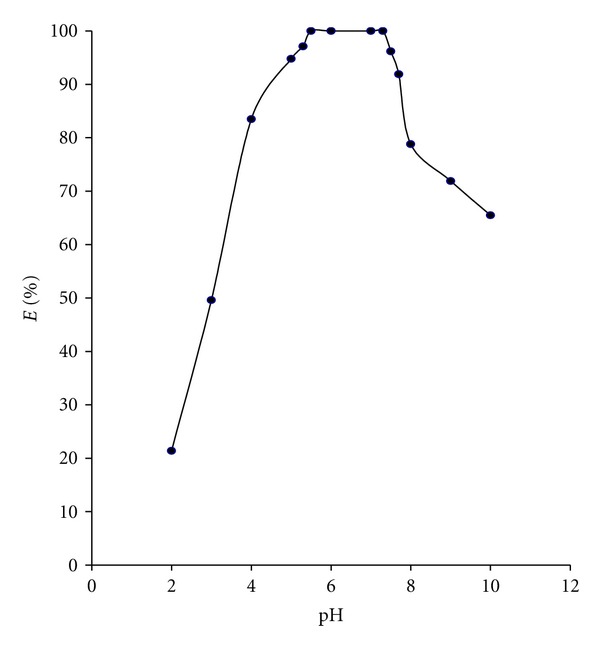
Extraction behaviour of neodymium(III) as a function of pH at 0.06 M sodium succinate; 0.1 M N-*n*-octylaniline in xylene.

The extraction of neodymium(III) was ascertained by carrying out the pH range between pH 1–10 with a 0.1 M solution of N-*n*-octylaniline in xylene at fixed concentration of 0.06 M sodium succinate. The pH for the quantitative extraction was 5.5–7.3 with N-*n*-octylaniline. Hence, the extraction of neodymium(III) was carried out at pH 6.0 for further studies (Figure 1).

### 3.2. Effect of the N-n-Octylaniline Concentration

Extraction of neodymium(III) was carried out by varying the reagent concentration from 0.005–0.5 M of N-*n*-octylaniline in xylene. It was observed that the extraction increased with increases in the concentration of extractant. The obtained result is shown in Table 1. Therefore, the optimum concentration of the extractant selected was 0.1 M N-*n*-octylaniline in xylene.

### 3.3. Effect of Weak Organic Acid Concentration

The extraction of neodymium(III) was examined at pH 6.0 with 0.1 M N-*n*-octylaniline in xylene in the presence of varying concentrations from 0.005 to 0.1 M of various weak organic acids (Table 2). The extraction of ion-pair complex of neodymium(III) was found to be quantitative in the range of 0.05–0.07 M sodium succinate. Hence, 0.06 M concentration of sodium succinate was used for further studies. While incomplete extraction of neodymium(III) was found to be in sodium salicylate, sodium malonate, and ascorbic acid media.

### 3.4. Effect of Diluents

Various aromatic and aliphatic organic solvents were studied as diluents for N-*n*-octylaniline (Table 3). It was found that the extraction of neodymium(III) was quantitative only with benzene and xylene as diluents for N-*n*-octylaniline. Xylene was preferred as diluent, because it provides better phase separation. Hence, 0.1 M N-*n*-octylaniline in xylene was used in all the studies.

### 3.5. Effect of Equilibration Time

**Figure 2:**
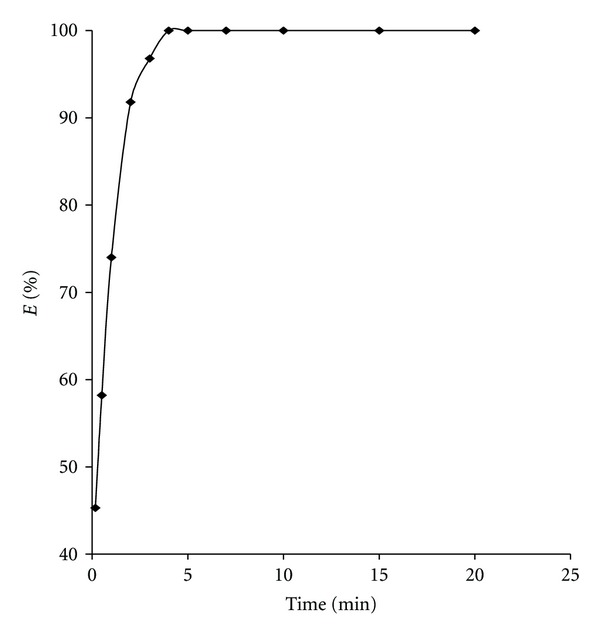
Effect of equilibration time on the extraction of neodymium(III) from 0.06 M sodium succinate at pH 6.0; 0.1 M N-*n*-octylaniline in xylene.

The extraction of neodymium(III) was studied for various periods of equilibration from 0.16 to 20 min with N-*n*-octylaniline. It was observed that, under the given experimental conditions, a minimum 4 min time was required for attaining the imitating extraction. It was checked that prolonged shaking time does not affect the extraction of neodymium(III). Hence, in all other studies the phase was equilibrated for 5 min (Figure 2).

### 3.6. Nature of Extracted Species

**Figure 3:**
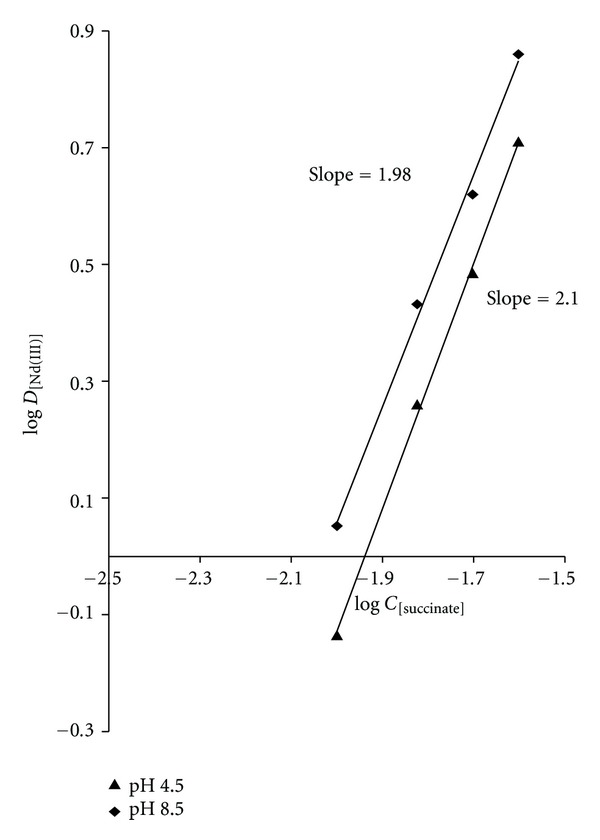
Log-log plot of log D_[Nd(III)]_versus log C_[Succinate]_ at 0.1 M N-*n*-octylaniline with pH 4.5 and pH 8.5.

**Figure 4:**
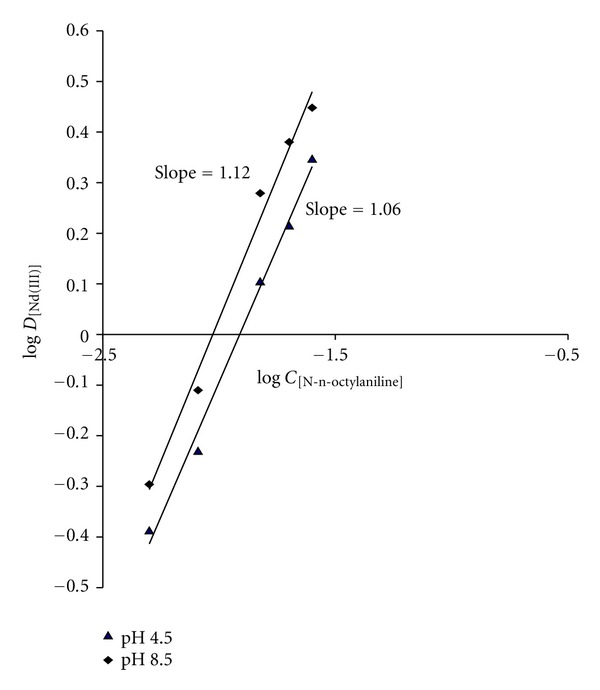
Log-log plot of log D_[Nd(III)]_ versus log C_[N-*n*-octylaniline]_ at 0.06 M sodium salicylate with pH 4.5 and pH 8.5.

An attempt was made to ascertain the nature of extracted species of neodymium(III) with the extractant using slope-ratio method. The distribution ratio of neodymium(III) evaluated at different concentration in molar of succinate was used at fixed N-*n*-octylaniline concentration at pH 4.5 and pH 8.5. A graph of log D_[Nd(III)]_ versus log C_[succinate]_ gave a slope of 1.98 and 2.10, respectively, (Figure 3). Similarly, a plot of log log D_[Nd(III)]_ versus log C_[N-*n*-octylaniline]_ at a fixed pH 4.5 and pH 8.5 with 0.06 M succinate gave slope of 1.06 and 1.12, respectively, (Figure 4). This indicates a mole ratio of neodymium(III) to succinate acid as 1 : 2 and that of N-*n*-octylaniline as 1 : 1. Thus, the extracted species was calculated to be an ion association complex with the probable composition 1 : 2 : 1 (metal : acid : extractant). The probable mechanism of extracted species is as follows:

(1) $$\begin{array}{c} {\text{RR}}^{\prime }{\text{NH}}_{\left(\text{org}\right)}+{{\text{H}}^{+}}_{\left(\text{aq}\right)}={\text{RR}}^{\prime }{{\text{N}{\text{H}}_{2}}^{+}}_{\left(\text{org}\right)}, \end{array}$$

(2) $$\begin{array}{c} {{\text{Nd}}^{3+}}_{\left(\text{aq.}\right)}+2\;{{\text{succinate}}^{2-}}_{\left(\text{aq.}\right)}={{\left[\text{Nd}{\left(\text{succinate}\right)}_{2}\right]}^{-}}_{\left(\text{aq.}\right)}, \end{array}$$

(3) $$\begin{array}{c} \begin{array}{cc} & {\text{RR}}^{\prime }{{{\text{NH}}_{2}}^{+}}_{\left(\text{org}\right)}+{{\left[\text{Nd}{\left(\text{succinate}\right)}_{2}\right]}^{-}}_{\left(\text{aq.}\right)}\\ & \;={\left[{\text{RR}}^{\prime }{{\text{NH}}_{2}}^{+}\text{Nd}{{\left(\text{succinate}\right)}_{2}}^{-}\right]}_{\left(\text{org}\right)}. \end{array} \end{array}$$

### 3.7. Effect of Stripping Agent

Various strong acids such as hydrochloric acid, nitric acid, and perchloric acid sulphuric acid in the range of 0.01–0.2 M were investigated as stripping agents for neodymium(III). The back-extraction or recovery from the organic phase achieved with perchloric acid was better from 0.09 to 0.12 M after 5 min shaking (Table 4). In this work 2 × 5 mL portions of 0.1 M perchloric acid were used as a suitable strippant.

### 3.8. Effect of Diverse Ions

The effect of various cations and anions on the recovery of neodymium(III) was investigated. The tolerance limit was set as the amount of foreign ions causing a change ±2% error in the recovery of neodymium(III). Initially the foreign ion was added to the neodymium(III) solution in large excess, 100 mg for anions, and 25 mg for cations. However, interference due to vanadium(V) and zirconium(IV) was eliminated by masking with 3 mg of F^−^. Manganese(V) was masked with 5 mg of oxalate. The anions such as EDTA and citrate were interfered in the extraction of neodymium(III) by the proposed method (Table 5).

## 4. Applications

### 4.1. Separation and Determination of Neodymium(III) from Binary Mixture

The separation of neodymium(III) from some commonly associated metal ions like U(VI), Th(IV), Nb(V), Zr(IV), Y(III), La(III), and Ce(IV) using N-*n*-octylaniline was achieved by taking advantage of the difference in the extraction conditions of metal such as pH of the aqueous phase, reagent concentration and use of masking agent (Table 6).

 Neodymium(III) was separated from U(VI), Th(IV), Nb(V), Zr(IV), and La(III) by its extraction with 10 mL of 0.1 M N-*n*-octylaniline in xylene from 0.06 M sodium succinate. Under this condition, the added metal ions remained quantitatively in the aqueous phase. The aqueous phase was washed with 5 mL xylene to remove traces of the reagent. Metal ions from the aqueous phase were determined by standard methods [20, 23–25] while neodymium(III) was determined by the proposed method after backstripping the organic phase.

 Similarly neodymium(III) was separated from Ce(IV) and Y(III) by masking with 10 mg fluoride and 50 mg thiosulphate, respectively. The masked Ce(IV) and Y(III) remained in the aqueous phase quantitatively under the optimum extraction condition of neodymium(III). After demasking of Ce(IV) and Y(III) with 5 mL hydrochloric acid, the solution was evaporated to dryness. They were determined spectrophotometrically with Arsenazo III at 660 nm [24] and Alizarin Red S [20], respectively.

### 4.2. Determination of Neodymium(III) in Synthetic Mixture

The applicability of the developed method for separation and determination of neodymium(III) in complex mixtures was studied (Table 7). In the synthetic mixtures, neodymium(III) was extracted under the optimum extraction conditions and quantitatively recovered in all mixtures.

## 5. Conclusion

- Quantitative extraction of neodymium(III) was achieved in 5 min with 0.1 M N-*n*-octylaniline in xylene at pH 6.0.
- The proposed extractive separation method is simple, rapid, selective, reproducible, and suitable for separation and determination of neodymium(III) from associated metal ions and synthetic mixtures.
- Trace level of neodymium(III) extracted using low concentration of N-*n*-octylaniline.
- The extraction mechanism corresponds to an anion exchange, in which a complex of stoichiometric formula [RR′NH_2_^+^Nd(succinate)_2_^−^]_org_ is formed in the organic phase.
- It is free from the interference of a large number of foreign ions.
- The solvent-like xylene showed good results for N-*n*-octylaniline compared with other diluents used.
- N-*n*-octylaniline can be synthesized at low cost, with high yield and best purity.

## Figures and Tables

**Table 1:** Extraction behavior of neodymium(III) as a function of N-*n*-octylaniline concentration. Nd(III) = 60 *μ*g, sodium succinate = 0.06 M, pH = 6.00 Aq : Org = 25 : 10, strippant = 0.1 M HClO_4_ (2 × 5 mL), equilibrium time = 5 min.

N-*n*-octylaniline (*M*)	Percentage extraction (% *E*)	Distribution ratio (*D*)
0.00	0.00	0.00
0.005	34.4	1.31
0.01	43.4	1.92
0.02	67.1	5.10
0.03	74.6	7.34
0.05	81.0	10.65
0.07	91.8	27.99
0.08	97.7	106.19
0.09	100	∞
0.1	100	∞
0.2	100	∞
0.3	100	∞
0.5	100	∞

**Table 2:** Extraction behaviour of neodymium(III) as a function of organic acid concentration. Nd(III) = 60 *μ*g, pH = 6.0, N-*n*-octylaniline = 0.1 M in xylene (10 mL), Aq : Org = 25 : 10, strippant = 0.1 M HClO_4_ (2 × 5 mL), equilibrium time = 5 min.

Acid (*M*)	Sodium salicylate		Sodium succinate		Sodium malonate		Ascorbic acid	
	%*E* ^a^	*D* ^b^	%*E* ^a^	*D* ^b^	%*E* ^a^	*D* ^b^	%*E* ^a^	*D* ^b^
0.005	13.6	0.39	24.6	0.815	20.0	0.63	9.3	0.25
0.01	33.0	1.23	42.6	1.85	31.0	1.12	17.5	0.47
0.02	47.2	2.23	57.4	3.36	52.8	2.80	27.0	0.93
0.03	57.1	3.33	82.0	11.38	61.7	4.03	36.8	1.45
0.04	67.2	5.12	94.8	45.57	78.0	8.86	46.7	2.19
0.05	83.2	12.38	100	∞	85.0	9.66	61.7	4.03
0.06	88.4	19.10	100	∞	89.0	20.23	69.3	5.64
0.07	85.2	14.39	100	∞	92.5	30.83	77.4	8.56
0.08	81.4	10.94	97.1	83.7	83.2	12.38	83.5	12.65
0.09	78.6	9.18	96.2	62.30	76.8	8.27	63.5	4.35
0.1	72.2	6.49	92.5	30.83	75.1	7.54	58.8	3.56
0.2	66.3	4.92	83.2	12.38	67.5	5.19	53.0	2.82

^a^Percentage extraction, ^b^distribution ratio.

**Table 3:** Extraction behaviour of neodymium(III) as a function of diluents. Nd(III) = 60 *μ*g, sodium succinate = 0.06 M, pH = 6.0, Aq : Org = 25 : 10, strippant = 0.1 M HClO_4_ (2 × 5 mL), and equilibrium time = 5 min.

Solvent	Dielectric constant, *ε*	Percentage extraction (%*E*)	Distribution ratio (*D*)
Benzene	2.28	100	∞
Toluene	2.38	89.0	20.22
Xylene	2.30	100	∞
Kerosene	1.80	63.5	4.34
Carbon tetrachloride	2.24	82.3	11.62
Chloroform	4.40	89.9	22.25
Amyl alcohol	13.90	74.8	8.82
1,2-Dichloroethane	10.5	68.4	5.41
MIBK	13.11	69.6	5.70
*n*-Butanol	17.8	81.4	10.94

**Table 4:** Extraction behaviour of neodymium(III) as a function of stripping agents. Nd(III) = 60 *μ*g, sodium succinate = 0.06 M, pH = 6.0, equilibrium time = 5 min, Aq. : Org = 25 : 10 N-*n*-octylaniline = 0.1 M in xylene, strippant = (2 × 5 mL).

Acid	HCl	HClO_4_	HNO_3_	H_2_SO_4_
*M*	Recovery (%)	Recovery (%)	Recovery (%)	Recovery (%)
0.005	47.0	65.7	55.8	34.9
0.01	54.6	73.2	57.9	42.6
0.03	67.5	80.5	68.6	49.3
0.05	69.3	89.3	79.7	63.7
0.07	76.7	98.3	80.3	62.2
0.09	80.3	100	81.0	60.1
0.1	82.2	100	85.9	55.9
0.15	87.3	100	74.8	53.9
0.2	85.7	100	72.0	47.8
0.3	80.2	92.6	67.3	43.6
0.5	73.5	83.9	65.9	29.3
0.7	67.9	78.8	61.2	27.4
1.0	63.5	75.3	57.4	23.5

Water-recovery 62.5%, acetate buffer (1) (pH = 3.42): recovery 55.8%,(2) (pH = 5.57): recovery 63.4%.

**Table 5:** Effect of various diverse ions. Nd(III) = 60 *μ*g pH = 6.00, sodium succinate = 0.06 M, Aq: Org = 25 : 10, N-*n*-octylaniline = 0.1 M in xylene (10 mL), equilibrium time = 5 min, and strippant = 0.1 M HClO_4_ (2 × 5 mL).

Ratio of added ions Nd(III): Ions	Amount tolerated (mg)	Foreign ion
1 : 833	50	Bromide, oxalate, citrate
1 : 417	25	Thiosulphate, acetate, thiourea, tartrate, Ca(II)
1 : 250	15	Nitrate, iodide
1 : 167	10	Malonate, ascorbate, fluoride, Sr(II), Mg(II)^b^, Cr(VI), Sm(III)
1 : 117	7	Phosphate, nitrite, W(VI), Cd(II), V(V)^a^, Pd(II)
1 : 83	5	Ti(IV), Y(III)^c^, Nb(V), Zr(IV)^a^
1 : 50	3	Gd(III)
1 : 33	2	Ba(II), Zn(II), Co(II)
1 : 16	1	Mo(II), Mn(II)
1 : 8	0.5	Fe(II), Ta(V), Ce(IV), La(III)
1 : 5	0.3	Ni(II), U(VI)

^a^Masked by 3 mg F^−^, ^b^masked by 5 mg oxalate, ^c^masked by 50 mg S_2_O_3_^−  −^.

**Table 6:** Separation of neodymium(III) from binary mixtures.

Metal ion	Metal ion added, (*μ*g)	Average (%) recovery*	Chromogenic ligand
Nd(III)	75	99.6	PAR
U(VI)	50	98.7	PAR
Nd(III)	75	99.8	
Zr(IV)	150	99.7	Alizarin Red S
Nd(III)	75	98.5	
Nb(V)	50	98.6	PAR
Nd(III)	75	99.8	
La(III)	60	99.5	Arsenazo I
Nd(III)	75	99.7	
Th(IV)	50	99.5	Arsenazo III
Nd(III)	75	99.7	
Ce(IV)^a^	50	99.5	Arsenazo III
Nd(III)	75	99.6	
Y(III)^b^	150	99.5	Alizarin Red S

^a^Masked with 10 mg fluoride, ^b^masked with 50 mg thiosulphate, and ^∗^average of six determinations.

**Table 7:** Separation of neodymium(III) from synthetic mixture.

Composition, (*μ*g)	Average (%) recovery*	Relative error, (%)
Nd(III) 60; U(VI) 50; Th(IV) 50	99.6	0.4
Nd(III)60; La(III) 60; Zr(IV) 150	99.5	0.5
Nd(III)60; Nb(V) 50; Zr(IV) 150	99.7	0.3
Nd(III)60; Y(III)^a^ 150; Nb(V) 50	99.5	0.5

^a^Masked with 50 mg thiosulphate, ^∗^average of five determinations.
